# Adaptive Tomotherapy for locally advanced unresectable pancreatic neuroendocrine tumor: Case report and literature review

**DOI:** 10.3389/fonc.2022.1045752

**Published:** 2022-11-14

**Authors:** Kuan-Yi Tu, Yen-Shuo Huang, Juntiong Lau, Hsin-Hua Lee

**Affiliations:** ^1^ School of Post Baccalaureate Medicine, Kaohsiung Medical University, Kaohsiung, Taiwan; ^2^ College of Medicine, Kaohsiung Medical University, Kaohsiung, Taiwan; ^3^ Department of Pathology, Kaohsiung Medical University Hospital, Kaohsiung Medical University, Kaohsiung, Taiwan; ^4^ Department of Surgery, Kaohsiung Medical University Hospital, Kaohsiung Medical University, Kaohsiung, Taiwan; ^5^ Ph.D. Program in Environmental and Occupational Medicine, Kaohsiung Medical University and National Health Research Institutes, Kaohsiung, Taiwan; ^6^ Department of Radiation Oncology, Kaohsiung Medical University Hospital, Kaohsiung, Taiwan; ^7^ Department of Radiation Oncology, Faculty of Medicine, College of Medicine, Kaohsiung Medical University, Kaohsiung, Taiwan; ^8^ Center for Cancer Research, Kaohsiung Medical University, Kaohsiung, Taiwan

**Keywords:** Tomotherapy, pancreas, abdomen, unresectable neuroendocrine tumor (NET), neuroendocrine neoplasm (NEN), image-guided radiotherapy (IGRT), adaptive planning, case report

## Abstract

**Background:**

Pancreatic neuroendocrine tumor (NET) is rare, and the majority presents late in their clinical course. Here, we present a huge locally advanced pancreatic NET having Hi-Art helical Tomotherapy that resulted in a 68% reduction in target volume during adaptive image-guided radiotherapy (IGRT).

**Case summary:**

A 63-year-old man without any history of systemic disease developed voiding difficulty for several months. Associated symptoms included poor appetite, nausea, distended abdomen, and body weight loss. Further magnetic resonance imaging showed a large multilobulated tumor in the left upper abdomen. Tumor biopsy revealed well-differentiated, grade 2, neuroendocrine tumor. Complete resection was unattainable. Therefore, Lanreotide was prescribed initially. However, tumor progression up to the greatest diameter of 18 cm was noted on computed tomography 5 months later. Thus, he stopped Lanreotide and commenced on concurrent chemoradiotherapy (CCRT). With a total dose of 70 Gy in 35 fractions, we generated two adaptive treatment plans during the whole course. Laparoscopic subtotal pancreatectomy with spleen preservation was performed after neoadjuvant CCRT. It has been more than 3 years after IGRT, and he remains cancer free and reports no side effects during regular follow-ups.

**Conclusion:**

Tomotherapy caused tumor size reduction and hence facilitated surgical possibility for this originally unresectable pancreatic NET. Neoadjuvant IGRT incorporated with adaptive treatment planning enhanced delivery accuracy. In this case of pancreatic NET resistant to Lanreotide, inter-fractional tumor regression from 1910 to 605 cc (68%) was documented.

## Introduction

Pancreatic neuroendocrine tumor (NET) is a rare type of neuroendocrine neoplasm (NEN) that arises from endocrine cell in pancreatic tissue, accounting for only 3% of all pancreatic tumors (1). The majority of pancreatic NETs are non-functional without defined clinical syndrome or abnormal hormone profiles, and their presentation is often delayed until significant mass effect or distant metastasis (2). In this situation, curative surgical resection is often intricate. However, the role of surgical resection in the treatment of pancreatic NET is imperative. Hill et al. have investigated the impact of resection on overall survival. Resection of pancreatic NET was related to significantly improved survival in contrast with those patients who were recommended for surgery but did not undergo resection (114 *vs*. 35 month; *p*<0.01) (2).

Surgery was, however, not recommended in cases of giant size and small probability of complete resection. Combining different treatment modalities prior to definitive surgical intervention was hence applied. Radiotherapy used as a main treatment of primary pancreatic NET is novel and not often reported, although it has long been regarded as an approved treatment option in palliative symptom relief (3). Here, we present a case of locally advanced unresectable pancreatic NET who underwent neoadjuvant concurrent chemoradiation (CCRT) *via* Tomotherapy and subsequent surgical resection successfully. To the best of our knowledge, this is the first reported pancreatic NET who had 68% regression of target volume during IGRT of 70 Gy.

## Case description

**Figure 1:**
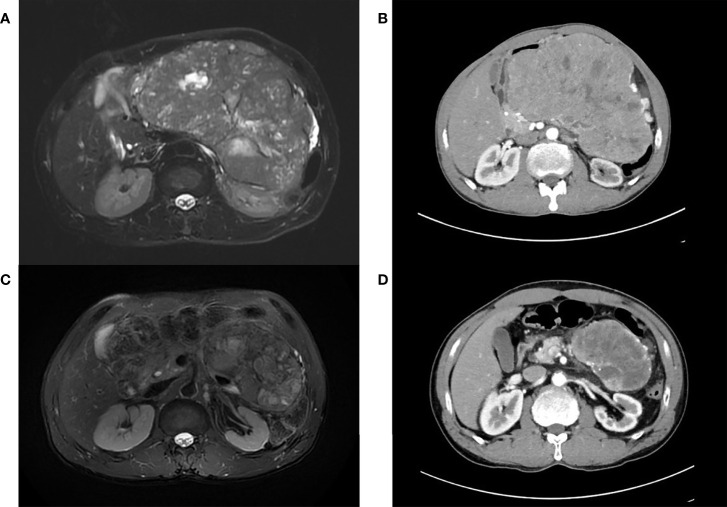
**(A)** Pretreatment magnetic resonance imaging (MRI) image depicting a multilobulated tumor with the size of 16.1 cm × 14.9 cm × 14.5 cm in the left upper abdomen. **(B)** Tumor progression up to the greatest diameter of 18 cm in the axial view of computerized tomography (CT) scans after treatment of Lanreotide. **(C)** Prominent tumor shrinkage after concurrent chemoradiation. **(D)** At least 50% reduction of tumor axial perpendicular diameters in preoperative CT.

**Figure 2:**
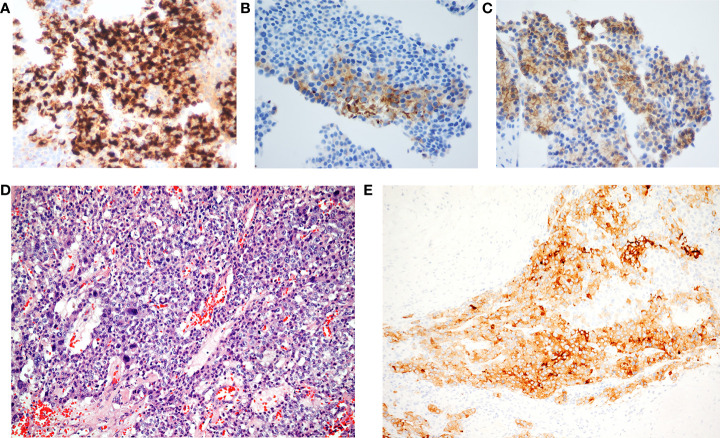
**(A)** Chromogranin, **(B)** synaptophysin, **(C)** somatostatin receptor 2A, and **(D)** monomorphous round to cuboidal cells arranging in solid and trabecular patterns. These cells have rich cytoplasm, and salt and pepper nuclei (hematoxylin–eosin stain; original magnification, 100×). **(E)** The immunohistochemical study reveals immunoreactivity of synaptophysin (original magnification, 100×).

A 63-year-old man without any history of systemic disease developed voiding difficulty for several months prior to his presence in the hospital. Associated symptoms included poor appetite, nausea, distended abdomen, and body weight loss. Further abdominal magnetic resonance imaging (MRI) showed a large multilobulated tumor with the size of 16.1 × 14.9 × 14.5 cm in the left upper abdomen (**Figure 1A**). During physical examination, a very big abdominal mass was palpated in the left upper quadrant with firm texture. The mass was fixed with regular border. His laboratory data, such as alpha-fetoprotein and carcinoembryonic antigen, were within normal limits. He received tumor biopsy in which it revealed tissue fragment infiltrated by tumor cells bearing relatively uniform round nuclei and high nucleus–cytoplasm ratio arranged in sheet or rosette-like patterns. Immunohistochemical staining showed positive for chromogranin A, synaptophysin, and somatostatin receptor 2A (SSTR2A) (**Figures 2A–C**). Moreover, the mitotic activity was about 3 per 10 high-power fields. The Ki-67 labeling index was about 4%. Well-differentiated, grade 2, neuroendocrine tumor was diagnosed.

There was no metastasis or regional lymph node involvement under initial MRI and further contrast-enhanced computerized tomography (CT). Because the tumor size was too large to differentiate the primary affected organ and the border of the tumor was implicated with surrounding organ, complete resection was not achievable. Therefore, Lanreotide was given initially. However, marked enlargement up to the greatest diameter of 18 cm was noted on CT scan 5 months later (**Figure 1B**). Coinciding with this, he complained about bulging abdomen interfering with digestion. In the second-line therapeutic regimens, there are multiple anti-tumor therapy including Everolimus- or Sunitinib-based targeted therapy, chemotherapy, and even peptide receptor radionuclide therapy (PRRT). Among these therapies, PRRT was not achievable in our hospital, and reimbursement for Temozolomide for pancreatic NET was not included in the National Health Insurance of our country. Given the bulky and progressive disease status, a second-line therapeutic regimen with cytotoxic chemotherapy was taken into consideration. After offering multidisciplinary treatment options in the full discussion with the patient and his families, CCRT was chosen for strengthening local control. Thus, he began to receive capecitabine + oxaliplatin (XELOX) concurrently with image-guided radiotherapy (IGRT).

**Figure 3:**
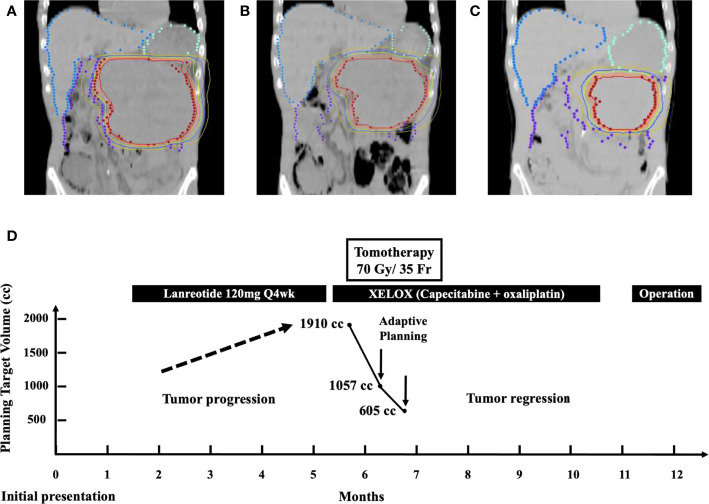
**(A)** Isodose curves depicting a target volume of 1,910 cc in the initial treatment plan of Tomotherapy. **(B)** Target volume reduced from 1,910 to 1,057 cc after 14 fractions. **(C)** Target volume reduced from 1,057 to 605 cc after 10 fractions. **(D)** Multidisciplinary treatment course of the patient from the day of initial presentation until definitive surgery: adaptive Tomotherapy plans were administrated twice at the timing of substantial target volume reduction from 1910 to 1,057 cc and from 1,057 to 605 cc, respectively.

We utilized the Hi-Art helical Tomotherapy, version 2.2.4.1 (TomoTherapy, Inc., Madison, WI). The planned total dose was 70 Gy in 35 fractions. The dose statistics was provided in the supplementary material regarding doses of the various organs at risk at each of the three plans, e.g., kidneys, liver, stomach, and spinal cord. After 14 fractions, we performed adaptive treatment planning to better suit regressed tumor. The target volume has shrunk from 1,910 to 1,057 cc (**Figures 3A, B**). Again, after 10 more fractions, IGRT revealed further shrinkage. Another new adaptive plan was administered, since the target volume has shrunk from 1,057 to 605 cc (**Figures 3B, C**). Corresponding with radiological response, his urinary and abdominal symptoms improved. The tumor volumes of the enhanced CT before and after radiotherapy have been calculated by the radiation oncologist utilizing a segmentation tool program. It was 2,199.35 cc before radiotherapy and 315.54 cc after radiotherapy. On the third month of CCRT, MRI showed a continuously decreased tumor dimension (**Figure 1C**). The axial perpendicular diameters of the tumor reduced at least 50% in preoperative CT after CCRT (**Figure 1D**).

His baseline performance status before CCRT was Eastern Cooperative Oncology Group (ECOG) grade 1 with only mild urinary frequency but no other complaint. There was also no acute radiation-induced nausea, diarrhea, or abdominal cramping. The radiotherapy-related toxicity was evaluated by the Common Terminology Criteria for Adverse Events (CTCAE) v4.0. Radiotherapy was well-tolerated without acute toxicities >2. In addition, his kidney function, as measured by creatinine clearance, remains mostly the same throughout the radiotherapy. He developed grade 1 radiation-induced dermatitis with mild erythema and later worsened because of a weekend trip swimming in the ocean. Grade 2 dermatitis subsided after Tomotherapy. Apart from avoiding disease progression, CCRT under current regimens resulted in tremendous tumor volume shrinkage. To achieve the best prognosis for the patient, curative surgical resection of tumor was indicated. Laparoscopic subtotal pancreatectomy with spleen preservation was performed, and the surgeons did not report any unusual difficulty. Pathology confirmed a pancreatic NET with the size of 12 × 10 × 6 cm and weighed 315.9 g with American Joint Committee on Cancer (AJCC) stage II, ypT3 (**Figure 2**). The surgical margin was 15 mm and uninvolved by tumor, which was confined to the pancreas.

The patient recovered well without post-operative complication. There was neither diabetes mellitus, postoperative ileus, nor surgery-related infection after subtotal pancreatectomy. Then, he was under regular surveillance in the outpatient department with abdominal CT every 3 months in the first year post-resection and every 12 months after the first year post-resection. It has been more than 46 months since his diagnosis of pancreatic NET, and he has no recurrence or distant metastasis during regular follow-ups. The overview of the clinical course of this patient is illustrated in **Figure 3D**.

## Discussion

Surgical resection is the only treatment that can cure pancreatic NETs, and it is recommended to remove all localized and limited metastatic disease (4). However, for our patient who presented with a sizeable tumor burden causing high surgical risk and impossibility of complete tumor resection, other modality like somatostatin analogues was applied. Within 5 months of commencing Lanreotide, this locally advanced tumor kept progressing. We shifted to CCRT for tumor growth control and symptom alleviation. Radiotherapy acted as a bridge forward to curative surgical resection. This patient was able to receive curative operation when the tumor became smaller to less than one-third of the original size after CCRT.

Although radiotherapy is not considered a curative method as much as surgery, Iwata et al. demonstrated that radiotherapy was effective for local control in pancreatic NET. This retrospective study included 11 patients with pancreatic NET who received radiotherapy with maximum dose of 60 Gy in 30 fractions and ended up having 100% disease control rate (1). In addition, symptomatic relief owing to reduction in the physical pressure from large tumor burden was obvious after radiotherapy. However, Iwata et al. disclosed that median progression-free survival (PFS) and median overall survival for patients with pancreatic NET were 5.5 months (95% confidence interval [CI], 3.7–28.2 months) and 35.9 months (95% CI, 9.04 months—not reached), respectively (1). On the contrary, our patient had been alive without disease recurrence for 45 months. In our case, radiotherapy was delivered up to 70 Gy in 35 fractions *via* Tomotherapy with image-guided and adaptive planning ability. Nine of 11 patients in the study of Iwata et al. utilized three-dimensional conformal radiation therapy, which delivered 50–54 Gy in 25–30 fractions (1). Moreover, the fact that 8 of 11 patients were in metastasis status and only one patient underwent post-RT surgical resection was the major cause of the different prognosis between the study of Iwata et al. and our case (1).

Tomotherapy allows radiation oncologists to visualize inter-fractional radiation responses. Kupelian et al. illustrated the benefit of IGRT in head and neck tumors by comparing the severity of inter-fractional setup errors. Even if imaging guidance was performed every other day, about 10% of all fractions still had a setup error over 5 mm (5). In addition to setup errors and organ mobilization, the dimension of targeted tumor was likely to change after each fraction, which may cause daily deviation. Different anatomic sites also have various setup uncertainties. Studies have shown that inter-fractional displacement in the lung is the largest followed by that in the abdomen (6). The mean 3D displacement (average of lateral, longitudinal, vertical, and rotational direction) of inter-fractional variation in the abdomen was 4.4 mm (6). The advantage of applying IGRT to avoid daily variation before abdominal irradiation is evident.

The recent advancements in radiotherapy technologies have made the delivery of the highly conformal dose to the target volume possible. The organ at risk that needed to be considered first was the kidneys. With the aim of maximal renal parenchymal sparing, radiotherapy was delivered. We provide the dose statistics in the supplementary material regarding the doses of the various organs at risk at each of the three plans, e.g., kidneys, liver, stomach, and spinal cord. With daily IGRT *via* Tomotherapy, we have carefully prescribed 70 Gy in 35 fractions, exceeding the doses of previous reports for pancreatic NET while causing no chronic-radiation-induced side effect (7). Bresciani et al. reported minimal toxicities from 66 Gy in 30 fractions delivered *via* Tomotherapy for para-aortic lymphadenopathy in the upper abdomen (8). The majority of patients received 45–50.4 Gy in 1.8 Gy per fraction as their abdominal radiation course. Radiation-induced diarrhea or emesis is commonly seen during abdominal–pelvic radiotherapy. Wang et al. has calculated that mean dose to the small bowel is associated with radiation-induced emesis. They suggested to limit the constraint of the small bowel mean dose to <63% of the prescribed dose (median, 28.35 Gy) (9). In the present case, the mean dose of the small bowel was 26.56, 24.97, and 18.39 Gy, respectively, in all three plans.

Somatostain analogues (SSAs) have been used in advanced or grade 1 or 2 (Ki-67 <10%) enteropancreatic, somatostatin receptor-positive NET (10), and the National Comprehensive Cancer Network (NCCN) guidelines have regarded it as an appropriate drug for symptom control and prevention of tumor progression (3). However, the huge tumor remained enlarged and resistant to Lanreotide in our case. The clinically predictive factors for tumor resistance in SSA can be determined by baseline tumor growth rate, and the patients can then be stratified by disease status and documented progression status to individualized treatment protocol (11). In pathological or molecular aspect, the NCCN guideline recommended using SSA on patients with positive SSTR2A expression (3). Recent research also showed the significant correlation between SSTR2A expression and the clinical efficacy of Lanreotide (12). Increased Ki-67 index and poorly differentiated NET also had unsatisfying SSA treatment outcome (13). In addition, genetic difference had been found between poorly differentiated neuroendocrine carcinoma (NEC) and well-differentiated NET. Poorly differentiated NEC, which includes small- and large- cell NEC, has frequent loss of immunolabeling patterns in p53 and Rb (14). On the other hand, loss of nuclear death domain-associated protein (DAXX) and alpha-thalassemia X-linked intellectual disability syndrome (ATRX) immunolabeling was observed in 5 of 11 (45%) well-differentiated pancreatic NET (14). There is also more clinical value of the finding of ATRX and DAXX gene mutations. In one recent meta-analysis, altered ATRX and DAXX gene had significant correlation with the prognosis of pancreatic NET (15). Disease- and relapse-free survival significantly decreased in patients who had ATRX and DAXX mutations (15). However, the present case did not undergo genetic testing as part of the assessment. More advanced investigation on molecular characteristics of pancreatic NET can help us predict the prognosis and set individualized clinical practice.

Our patient had a huge locally advanced abdominal tumor cured without chronic treatment-related complication. We searched on PubMed and Medline databases for articles written in English from 2016 to 2022 with keywords such as “Abdominal mass,” “Neuroendocrine tumor,” and “locally advanced.” **Table 1** shows the clinicopathological characteristics of eight recent cases. The definitive treatment for NET is surgical resection, and the resectability is associated with size and location. The cases whose primary site was in the liver or duodenum or colon received surgery as primary treatment even if the tumor size was up to 20 cm × 16 cm × 11 cm (18–20, 22). Tumors with the pancreas as the primary site and with an initial tumor diameter of 4/3.8 cm were able to be resected (16), and yet, some researchers preferred chemotherapy and radiotherapy in tumors with the greatest diameter up to 9.8 cm (17). In the case of a 9.8-cm pancreatic NET, systemic therapy followed by CCRT with 54 Gy in 30 fractions was applied and reached partial response (17). Similar to our case, CCRT was used. With unprecedented 70 Gy, a 68% reduction in target volume followed by a successful conversion to resectable status was presented in our case. Quite the opposite, colorectal NET was rare and often diagnosed very late, and bowel perforation was noted in the case of Alshammari et al. (19). Namikawa et al. presented a case of gastric NET that developed hepatic metastasis (diameter up to 25 cm). Spontaneous rupture of hepatic metastasis was noted 8 months after initial treatment with everolimus plus somatostatin (21).

**Table 1:** Clinicopathological characteristics of reported cases of locally advanced abdominal neuroendocrine tumor.

Articles	Age (year)	Sex	Location	Grade (G)	Initial size (cm)	Treatment 1	Treatment 2	Treatment 3	Outcome
Mirică et al., 2016 (16)	59	F	Pancreas	G2	4/3.8	Surgery	Somatostatin analogue	Chemotherapy	^#^Alive 34 months
Won et al., 2017 (17)	52	F	Pancreas	NEC	9.8	Etoposide + cisplatin	CCRT (etposide+. Cisplatin + 54 Gy/30 Fr)	Irinotecan + cisplatin	^§^Alive 11 months
Meng et al., 2018 (18)	56	F	Liver	G1	20×16×11	Surgery	None	None	^§^Alive 6 years
Alshammari et al., 2019 (19)	57	M	Colon	NEC	9×7	Surgery	None	None	N/A
Wang et al., 2021 (20)	55	F	Duodenum	G2	6.2×5.8	Surgery	None	None	^§^Alive 3 months
Namikawa et al., 2021 (21)	64	M	Stomach	G3	*25	Everolimus + somatostatin	None	None	*Died 8 months
Felux et al., 2022 (22)	65	F	Colon	NEC	9.8–10.5	Surgery	Carboplatin + Etoposide	None	N/A
Present case, 2022	63	M	Pancreas	G2	16.1× 14.9× 14.5	Lanreotide	CCRT (XELOX+ 70 Gy/35 Fr)	Surgery	^§^Alive 45 months

F, female; M, male; NEC, neuroendocrine carcinoma; CCRT, concurrent chemoradiotherapy; Gy, Gray; Fr, fraction; XELOX, Capecitabine plus oxaliplatin; N/A, not applicable.

*The case was diagnosed with hemoperitoneum due to hemorrhaging of the enormous liver metastasis with 25 cm in diameter. CT imaging revealed progression of liver metastasis, and the patient died 8 months after initial treatment.

^#^Alive since diagnosis. ^§^Alive since initial treatment.

Further prospective studies with larger patient numbers are required to establish the role of IGRT in huge NET (7). However, it is often not expected to see one with such considerable size in the present case. It is imperative to take into consideration various treatment options for the best benefits of each individual patient. As in our case, IGRT showed its value in optimizing the therapeutic ratio by maximizing target dose safely. Most of all, the conversion of surgical suitability has extended his disease-free survival.

## Conclusions

IGRT *via* Tomotherapy has eased the patients’ symptoms from such 18-cm pancreatic NET in this case. Apart from complete relief of abdominal and pelvic discomfort, CCRT caused a 68% target volume reduction (1,910 to 605 cc) and facilitated further surgical resectability. To the best of our knowledge, this is the first reported case using Tomotherapy to deliver 70 Gy to a pancreatic NET with such favorable outcome. Adaptive planning helps to modify doses according to volumetric changes.

## Patient perspective

I was at first disheartened with the diagnosis of an inoperable tumor. When I came to the Department of Radiotherapy, I was dismayed and yet impressed by the coordination of simulation scanning and resource intensive re-planning due to markedly shrinkage of the tumor. Following more and more fractions of Tomotherapy, I felt vigorous because of a flatter belly coinciding with the diminished tumor volume. I remember swimming at the beach during the radiation treatment course, and the belly skin became painful, which was later relieved by topical medication from Dr. Lee. She advised me to be heedful of skin care. Owing to the strikingly smaller size after radiotherapy, I was able to take on surgery followed by an uneventful postoperative recovery. It has been almost 4 years, and I am energetic with my cancer-free life.

## Data availability statement

The original contributions presented in the study are included in the article/**Supplementary Material**. Further inquiries can be directed to the corresponding author.

## Ethics statement

Ethical review and approval was not required for the study on human participants in accordance with the local legislation and institutional requirements. The patients/participants provided their written informed consent to participate in this study. Written informed consent was obtained from the individual(s) for the publication of any potentially identifiable images or data included in this article.

## Author contributions

K-YT and JL wrote the first draft of the manuscript and made the table. K-YT and Y-SH contributed to image review and figure legends. K-YT generated the timeline figure. H-HL treated the patient, conceived the paper layout, and revised the manuscript. All authors contributed to the article and approved the submitted version.

## Conflict of interest

The authors declare that the research was conducted in the absence of any commercial or financial relationships that could be construed as a potential conflict of interest.

## Publisher’s note

All claims expressed in this article are solely those of the authors and do not necessarily represent those of their affiliated organizations, or those of the publisher, the editors and the reviewers. Any product that may be evaluated in this article, or claim that may be made by its manufacturer, is not guaranteed or endorsed by the publisher.

## References

[1] Iwata T Ueno H Itami J Ito Y Inaba K Morizane C . Efficacy of radiotherapy for primary tumor in patients with unresectable pancreatic neuroendocrine tumors. Jpn J Clin Oncol (2017) 47(9):826–31. doi: 10.1093/jjco/hyx081 28591817

[2] Hill JS McPhee JT McDade TP Zhou Z Sullivan ME Whalen GF . Pancreatic neuroendocrine tumors: The impact of surgical resection on survival. Cancer (2009) 115(4):741–51. doi: 10.1002/cncr.24065 19130464

[3] Shah MH Goldner WS Benson AB Bergsland E Blaszkowsky LS Brock P . Neuroendocrine and adrenal tumors, version 2. 2021 Nccn Clin Pract Guidelines Oncol J Natl Compr Canc Netw (2021) 19(7):839–68. doi: 10.6004/jnccn.2021.0032 34340212

[4] Akirov A Larouche V Alshehri S Asa SL Ezzat S . Treatment options for pancreatic neuroendocrine tumors. Cancers (Basel) (2019) 11(6):828. doi: 10.3390/cancers11060828 31207914 PMC6628351

[5] Kupelian P Langen K . Helical tomotherapy: Image-guided and adaptive radiotherapy. Front Radiat Ther Oncol (2011) 43:165–80. doi: 10.1159/000322420 21625153

[6] Zhou J Uhl B Dewit K Young M Taylor B Fei DY . Analysis of daily setup variation with tomotherapy megavoltage computed tomography. Med Dosim (2010) 35(1):31–7. doi: 10.1016/j.meddos.2009.01.005 19931012

[7] Chan DL Thompson R Lam M Pavlakis N Hallet J Law C . External beam radiotherapy in the treatment of gastroenteropancreatic neuroendocrine tumours: A systematic review. Clin Oncol (R Coll Radiol) (2018) 30(7):400–8. doi: 10.1016/j.clon.2018.03.006 29615284

[8] Bresciani S Garibaldi E Cattari G Maggio A Di Dia A Delmastro E . Dose to organs at risk in the upper abdomen in patients treated with extended fields by helical tomotherapy: A dosimetric and clinical preliminary study. Radiat Oncol (2013) 8:247. doi: 10.1186/1748-717x-8-247 24160769 PMC3816584

[9] Wang YM Chen YF Lee PY Ho MW Huang EY . Radiation-induced emesis (Rie) in extended-field radiotherapy for gynecological malignancies: Dosimetric and non-dosimetric factors. Curr Oncol (2021) 28(5):3602–9. doi: 10.3390/curroncol28050308 PMC8482175 34590609

[10] Caplin ME Pavel M Ćwikła JB Phan AT Raderer M Sedláčková E . Lanreotide in metastatic enteropancreatic neuroendocrine tumors. N Engl J Med (2014) 371(3):224–33. doi: 10.1056/NEJMoa1316158 25014687

[11] Carmona-Bayonas A Jiménez-Fonseca P Lamarca Á Barriuso J Castaño Á Benavent M . Prediction of progression-free survival in patients with advanced, well-differentiated, neuroendocrine tumors being treated with a somatostatin analog: The getne-trasgu study. J Clin Oncol (2019) 37(28):2571–80. doi: 10.1200/jco.19.00980 PMC6768612 31390276

[12] Kasajima A Papotti M Ito W Brizzi MP La Salvia A Rapa I . High interlaboratory and interobserver agreement of somatostatin receptor immunohistochemical determination and correlation with response to somatostatin analogs. Hum Pathol (2018) 72:144–52. doi: 10.1016/j.humpath.2017.11.008 29180250

[13] Lee L Ramos-Alvarez I Jensen RT . Predictive factors for resistant disease with Medical/Radiologic/Liver-directed anti-tumor treatments in patients with advanced pancreatic neuroendocrine neoplasms: Recent advances and controversies. Cancers (Basel) (2022) 14(5):1250. doi: 10.3390/cancers14051250 35267558 PMC8909561

[14] Yachida S Vakiani E White CM Zhong Y Saunders T Morgan R . Small cell and Large cell neuroendocrine carcinomas of the pancreas are genetically similar and distinct from well-differentiated pancreatic neuroendocrine tumors. Am J Surg Pathol (2012) 36(2):173–84. doi: 10.1097/PAS.0b013e3182417d36 PMC3261427 22251937

[15] Wang F Xu X Ye Z Qin Y Yu X Ji S . Prognostic significance of altered Atrx/Daxx gene in pancreatic neuroendocrine tumors: A meta-analysis. Front Endocrinol (Lausanne) (2021) 12:691557. doi: 10.3389/fendo.2021.691557 34220718 PMC8253224

[16] Mirică A Bădărău IA Mirică R Păun S Păun DL . A rare case of metastasized non-functional pancreatic neuroendocrine tumor with a good long-term survival. J Med Life (2016) 9(4):369–72. doi: 10.22336/jml.2016.0409 PMC5141396 27928440

[17] Won YG Seo KJ Hyeon J Shin OR Chang E Sun S . Gastroenteropancreatic-origin neuroendocrine carcinomas: Three case reports with favorable responses following localized radiotherapy and a review of literature. Med (Baltimore) (2017) 96(49):e9009. doi: 10.1097/md.0000000000009009 PMC5728897 29245282

[18] Meng XF Pan YW Wang ZB Duan WD . Primary hepatic neuroendocrine tumor case with a preoperative course of 26 years: A case report and literature review. World J Gastroenterol (2018) 24(24):2640–6. doi: 10.3748/wjg.v24.i24.2640 PMC6021771 29962820

[19] Alshammari TF Hakami RA Alali MN AlShammari S Zayed MA AlSohaibani MO . A perforated colonic neuroendocrine tumor with liver metastasis: A case report and literature review. Am J Case Rep (2019) 20:920–5. doi: 10.12659/ajcr.916288 PMC6613493 31249283

[20] Wang X Wu Y Cao X Zhang X Cheng Y Kong L . Duodenal neuroendocrine tumor: A rare case report. Med (Baltimore) (2021) 100(6):e24635. doi: 10.1097/md.0000000000024635 PMC7886419 33578581

[21] Namikawa T Yokota K Yamaguchi S Fukudome I Munekage M Uemura S . Spontaneous intra-abdominal hemorrhage of a well-differentiated, grade 3 gastric neuroendocrine tumor during drug-based treatment. Clin J Gastroenterol (2021) 14(4):1244–9. doi: 10.1007/s12328-021-01433-3 33977396

[22] Felux K McCarty B Turner D Gray T Patel V . Poorly differentiated Large cell neuroendocrine carcinoma of the colon: A case report. Cureus (2022) 14(1):e20949. doi: 10.7759/cureus.20949 35154929 PMC8815286

