# Supplementary figures and images for: Adaptive Tomotherapy for locally advanced unresectable pancreatic neuroendocrine tumor: Case report and literature review
Source: Front Oncol. 2022 Nov 14;12:1045752. doi: 10.3389/fonc.2022.1045752 (PMC9702327; doi:10.3389/fonc.2022.1045752)

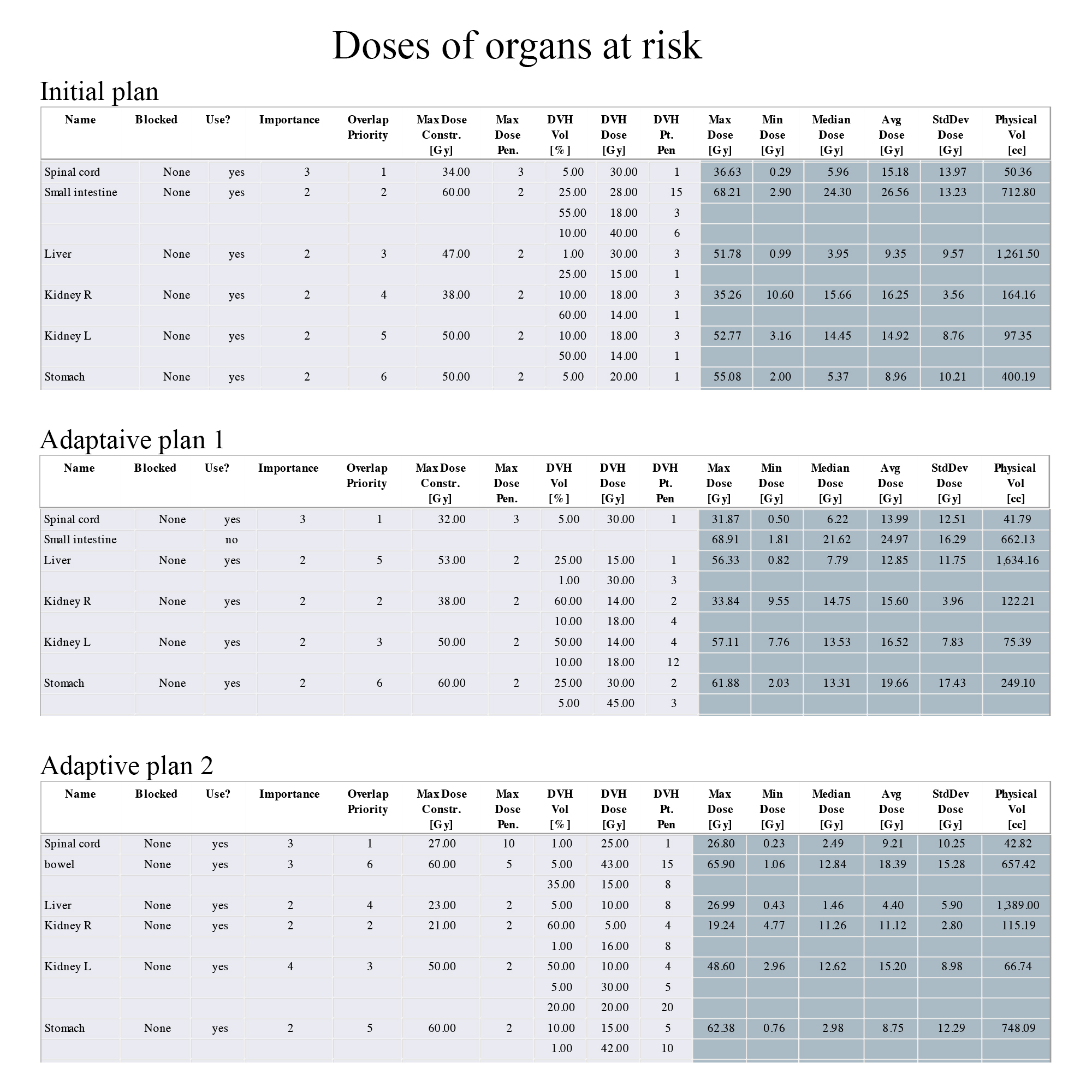

Supplement: Supplementary file 1 [file Image_1.jpeg]
